# Supplementary material for: Empirical and Computational Evaluation of Hemolysis in a Microfluidic Extracorporeal Membrane Oxygenator Prototype
Source: Micromachines (Basel). 2024 Jun 15;15(6):790. doi: 10.3390/mi15060790 (PMC11205701; doi:10.3390/mi15060790)
Supplement: Supplementary file 1 [file micromachines-15-00790-s001.zip › micromachines-2953850-supplementary.pdf]

Supplementary:

Table S1: One-way ANOVA p values, (cross-device comparison of empirical values), boxes highlighted in orange ($p < 0.05$) means the IHPP difference was statistically significant. Whereas the non-highlighted boxes represent the differences not to be statistically significant.

Devices	Negative Control	Baseline	Reduced Port	Reduced Gap	Herringbone
Negative Control	-	-	-	-	-
Baseline	>0.9999	-	-	-	-
Reduced Port	>0.9999	>0.9999	-	-	-
Reduced Gap	>0.9999	>0.9999	>0.9999	-	-
Herringbone	>0.9999	>0.9999	>0.9999	>0.9999	-
Positive Control	<0.0001	<0.0001	<0.0001	<0.0001	<0.0001

Table S2: One sample t-test p values, (empirical vs model), boxes highlighted in green ($p > 0.05$) means the difference between the IHPP of empirical and model was not statistically significant. Whereas the non-highlighted boxes represent statistically significant differences.

	Models								
Devices	1	2	3	4	5	6	7	8	9
Negative Control	<0.0001	<0.0001	<0.0001	0.0056	0.0034	0.0095	0.3538	0.1461	0.0056
Baseline	<0.0001	<0.0001	<0.0001	0.0029	0.002	0.0036	0.3261	0.0384	0.0237
Reduced Port	<0.0001	<0.0001	<0.0001	0.2413	0.0301	0.0028	<0.0001	<0.0001	<0.0001
Reduced Gap	<0.0001	<0.0001	<0.0001	0.0053	0.567	0.0001	<0.0001	<0.0001	<0.0001
Herringbone	<0.0001	<0.0001	<0.0001	0.0057	0.0031	0.0081	0.0216	0.2246	0.001
Positive Control	<0.0001	<0.0001	<0.0001	0.8388	0.0592	0.106	<0.0001	0.0001	<0.0001

Table S3: Device shear rates for 100 ml/min flow rate

Device	Volm. Avg. Shear Rate (1/s)	Max. Wall Shear Rate (1/s)
Negative Control	284	600
Baseline	363	37,400
Reduced Port	597	150,000
Reduced Gap	947	105,000
Herringbone	486	51,000
Positive Control	14,267	273,000

Table S4: Properties of each hemolysis model tested *in-silico.*

Model Name	Governing Equation	Shear Stress Model	Parameter Set	C	α	β
PL-1	$D(\tau,t)=\frac{\Delta fHb}{Hb}=C\tau^{\alpha}t^{\beta}$	τ_{vm}	Giersiepen	3.63×10^{-7}	2.416	0.785
PL-2	$D(\tau,t)=\frac{\Delta fHb}{Hb}=C\tau^{\alpha}t^{\beta}$	τ_{b}	Giersiepen	3.63×10^{-7}	2.416	0.785
PL-3	$D(\tau,t)=\frac{\Delta fHb}{Hb}=C\tau^{\alpha}t^{\beta}$	τ_{p}	Giersiepen	3.63×10^{-7}	2.416	0.785
PL-4	$D(\tau,t)=\frac{\Delta fHb}{Hb}=C\tau^{\alpha}t^{\beta}$	τ_{vm}	Heuser/Opitz	1.80×10^{-8}	1.991	0.765
PL-5	$D(\tau,t)=\frac{\Delta fHb}{Hb}=C\tau^{\alpha}t^{\beta}$	τ_{b}	Heuser/Opitz	1.80×10^{-8}	1.991	0.765
PL-6	$D(\tau,t)=\frac{\Delta fHb}{Hb}=C\tau^{\alpha}t^{\beta}$	τ_{p}	Heuser/Opitz	1.80×10^{-8}	1.991	0.765
PL-7	$D(\tau,t)=\frac{\Delta fHb}{Hb}=C\tau^{\alpha}t^{\beta}$	τ_{vm}	Zhang	1.228×10^{-7}	1.9918	0.6606
PL-8	$D(\tau,t)=\frac{\Delta fHb}{Hb}=C\tau^{\alpha}t^{\beta}$	τ_{b}	Zhang	1.228×10^{-7}	1.9918	0.6606
PL-9	$D(\tau,t)=\frac{\Delta fHb}{Hb}=C\tau^{\alpha}t^{\beta}$	τ_{p}	Zhang	1.228×10^{-7}	1.9918	0.6606
TH-1	$D(\tau,t)=\frac{\Delta fHb}{Hb}=\sum_{i=inlet}^{i} C\beta\left[\sum_{j=1}^{j}\tau_{t_j}^{\frac{\alpha}{\beta}}\Delta t_j\right]^{\beta-1}\times\tau_{t_i}^{\frac{\alpha}{\beta}}\Delta t_i$	τ_{vm}	Giersiepen	3.63×10^{-7}	2.416	0.785
TH-2	$D(\tau,t)=\frac{\Delta fHb}{Hb}=\sum_{i=inlet}^{i} C\beta\left[\sum_{j=1}^{j}\tau_{t_j}^{\frac{\alpha}{\beta}}\Delta t_j\right]^{\beta-1}\times\tau_{t_i}^{\frac{\alpha}{\beta}}\Delta t_i$	τ_{b}	Giersiepen	3.63×10^{-7}	2.416	0.785
TH-3	$D(\tau,t)=\frac{\Delta fHb}{Hb}=\sum_{i=inlet}^{i} C\beta\left[\sum_{j=1}^{j}\tau_{t_j}^{\frac{\alpha}{\beta}}\Delta t_j\right]^{\beta-1}\times\tau_{t_i}^{\frac{\alpha}{\beta}}\Delta t_i$	τ_{p}	Giersiepen	3.63×10^{-7}	2.416	0.785
TH-4	$D(\tau,t)=\frac{\Delta fHb}{Hb}=\sum_{i=inlet}^{i} C\beta\left[\sum_{j=1}^{j}\tau_{t_j}^{\frac{\alpha}{\beta}}\Delta t_j\right]^{\beta-1}\times\tau_{t_i}^{\frac{\alpha}{\beta}}\Delta t_i$	τ_{vm}	Heuser/Opitz	1.80×10^{-8}	1.991	0.765
TH-5	$D(\tau,t)=\frac{\Delta fHb}{Hb}=\sum_{i=inlet}^{i} C\beta\left[\sum_{j=1}^{j}\tau_{t_j}^{\frac{\alpha}{\beta}}\Delta t_j\right]^{\beta-1}\times\tau_{t_i}^{\frac{\alpha}{\beta}}\Delta t_i$	τ_{b}	Heuser/Opitz	1.80×10^{-8}	1.991	0.765
TH-6	$D(\tau,t)=\frac{\Delta fHb}{Hb}=\sum_{i=inlet}^{i} C\beta\left[\sum_{j=1}^{j}\tau_{t_j}^{\frac{\alpha}{\beta}}\Delta t_j\right]^{\beta-1}\times\tau_{t_i}^{\frac{\alpha}{\beta}}\Delta t_i$	τ_{p}	Heuser/Opitz	1.80×10^{-8}	1.991	0.765
TH-7	$D(\tau,t)=\frac{\Delta fHb}{Hb}=\sum_{i=inlet}^{i} C\beta\left[\sum_{j=1}^{j}\tau_{t_j}^{\frac{\alpha}{\beta}}\Delta t_j\right]^{\beta-1}\times\tau_{t_i}^{\frac{\alpha}{\beta}}\Delta t_i$	τ_{vm}	Zhang	1.228×10^{-7}	1.9918	0.6606
TH-8	$D(\tau,t)=\frac{\Delta fHb}{Hb}=\sum_{i=inlet}^{i} C\beta\left[\sum_{j=1}^{j}\tau_{t_j}^{\frac{\alpha}{\beta}}\Delta t_j\right]^{\beta-1}\times\tau_{t_i}^{\frac{\alpha}{\beta}}\Delta t_i$	τ_{b}	Zhang	1.228×10^{-7}	1.9918	0.6606
TH-9	$D(\tau,t)=\frac{\Delta fHb}{Hb}=\sum_{i=inlet}^{i} C\beta\left[\sum_{j=1}^{j}\tau_{t_j}^{\frac{\alpha}{\beta}}\Delta t_j\right]^{\beta-1}\times\tau_{t_i}^{\frac{\alpha}{\beta}}\Delta t_i$	τ_{p}	Zhang	1.228×10^{-7}	1.9918	0.6606
